# Orthogonal pinhole-imaging-based learning salp swarm algorithm with self-adaptive structure for global optimization

**DOI:** 10.3389/fbioe.2022.1018895

**Published:** 2022-12-01

**Authors:** Zongshan Wang, Hongwei Ding, Jingjing Yang, Peng Hou, Gaurav Dhiman, Jie Wang, Zhijun Yang, Aishan Li

**Affiliations:** ^1^ School of Information Science and Engineering, Yunnan University, Kunming, China; ^2^ University Key Laboratory of Internet of Things Technology and Application, Kunming, China; ^3^ School of Computer Science, Fudan University, Shanghai, China; ^4^ Department of Electrical and Computer Engineering, Lebanese American University, Byblos, Lebanon; ^5^ Department of Computer Science and Engineering, University Centre for Research and Development, Chandigarh University, Gharuan, India; ^6^ Department of Computer Science and Engineering, Graphic Era Deemed to be University, Dehradun, India; ^7^ School of Mechanical and Power Engineering, Zhengzhou University, Zhengzhou, China; ^8^ Rackham Graduate School, University of Michigan, Ann Arbor, MI, United States

**Keywords:** particle swarm optimization, exploration and exploitation, metaheuristic algorithms, equilibrium optimizer, global optimization, benchmark

## Abstract

Salp swarm algorithm (SSA) is a simple and effective bio-inspired algorithm that is gaining popularity in global optimization problems. In this paper, first, based on the pinhole imaging phenomenon and opposition-based learning mechanism, a new strategy called pinhole-imaging-based learning (PIBL) is proposed. Then, the PIBL strategy is combined with orthogonal experimental design (OED) to propose an OPIBL mechanism that helps the algorithm to jump out of the local optimum. Second, a novel effective adaptive conversion parameter method is designed to enhance the balance between exploration and exploitation ability. To validate the performance of OPLSSA, comparative experiments are conducted based on 23 widely used benchmark functions and 30 IEEE CEC2017 benchmark problems. Compared with some well-established algorithms, OPLSSA performs better in most of the benchmark problems.

## 1 Introduction

In recent years, metaheuristics have received incredible attention worldwide, and their great success on global optimization tasks has established superb beliefs for researchers, motivating them to develop more algorithms with good performance. Basically, metaheuristics are classified into four categories, namely swarm-based, human-based, evolution-based and physics-based approaches. Among them, swarm intelligence-based methods have attracted most enthusiastic admiration, and they usually metaphorically represent some unique swarming behavior of organisms in the nature. The most classical swarm intelligent algorithm, particle swarm optimization (PSO) algorithm (Kennedy and Eberhart, 1995), mimics the flocking behavior of birds as they fly through the sky. Artificial bee colony (ABC) algorithm (Karaboga and Basturk, 2007; Wang et al., 2020), inspired by the collaborative honey-harvesting behavior of bees, has also captured widespread attention and has been successfully applied to solve real-word problems. The ant colony optimization (ACO) algorithm (Blum, 2005) is motivated by the phenomenon that ant colonies transmit information by secreting pheromones to accomplish foraging. The ACO algorithm is admired by researchers because of its unique advantages in solving business travel problems. Besides, many excellent nature-inspired swarm intelligent approaches have been validated to be effective in tricky global optimization projects, they include but are not limited to: bat algorithm (BA) (Yang and Gandomi, 2012), krill herd optimization (KHO) (Gandomi et al., 2012), cuckoo search (CS) algorithm (Gandomi et al., 2013), fruit-fly optimization algorithm (FOA) (Mitić et al., 2015), grey wolf optimizer (GWO) (Mirjalili et al., 2014), moth-flame optimization (MFO) (Mirjalili, 2015), grasshopper optimization algorithm (GOA) (Abualigah and Diabat, 2020), whale optimization algorithm (WOA) (Mirjalili and Lewis, 2016), marine predators algorithm (MPA) (Faramarzi et al., 2020a), white shark optimizer (WSO) (Braik et al., 2022), starling murmuration optimizer (SMO) (Zamani et al., 2022), harris hawks algorithm (Heidari et al., 2019), squirrel search optimization (SSO) algorithm (Jain et al., 2019), dragonfly algorithm (DA) (Mirjalili, 2016), chimp optimization algorithm (ChOA) (Khishe and Mosavi, 2020), rat swarm algorithm (RSA) (Dhiman et al., 2021), Animal migration optimization (AMO) (Li et al., 2014), butterfly optimization algorithm (BOA) (Arora and Singh, 2019), emperor penguin optimizer (EPO) (Dhiman and Kumar, 2018), tunicate swarm algorithm (TSA) (Kaur et al., 2020), horse herd optimization algorithm (HOA) (MiarNaeimi et al., 2021), monarch butterfly optimization (MBO) (Wang et al., 2019), firefly algorithm (Fister et al., 2013; Wang et al., 2022a), and seagull optimization algorithm (SOA) (Dhiman and Kumar, 2019).

Swarm intelligent algorithms have emerged in various scientific and engineering fields, they are based on different metaphors, and their mathematical models consequently differ, which correspond to distinctive search mechanisms. Nevertheless, the framework of these algorithms is broadly the same, all divided into two phases: exploration (cohesion) and exploitation (alignment) (Wang et al., 2022b). In the exploration phase, it is encouraged to maximize the stochasticity of the search agents, which is related to the global search process. In the later iteration, the algorithm shifts from exploration to exploitation, refining the promising regions that have already been explored, which is pertinent to the local search process. Balancing these two phases is a core essential and challenging task for metaheuristic techniques.

Recently, a novel nature-inspired swarm intelligent technique, namely salp swarm algorithm (SSA) (Mirjalili et al., 2017), has been reported by Mirjalili in 2017. SSA simulates the distinctive foraging and navigation behaviors of the marine biological salps. The framework is mainly based on the leader-follower mechanism of the salp swarm. Compared with other population intelligence-based approaches, SSA has many advantages, such as fewer control parameters, easy implementation, and special search pattern. Prior studies have shown that SSA displays better performance than other metaheuristic techniques on numerical optimization problems and engineering design cases. Therefore, SSA is favored and employed to tackle various optimization problems. In (Ewees et al., 2021), Ewees et al. enhanced SSA algorithm using firefly search mechanism for solving unrelated parallel machine scheduling problem. In (Xia et al., 2022), Xia et al. proposed barebone SSA algorithm, and embedded quasi-oppositional based learning strategy. The developed SSA variant was used in medical diagnosis systems. In (Ozbay and Alatas, 2021), Ozbay et al. added inertia weights to the standard SSA to improve the ability of the algorithm to find the optimal solution and utilized the boosted SSA for fake news detection and obtained satisfactory results. In, Faris et al. (2018) introduced a binary version of SSA with a crossover mechanism for feature selection problems. In, Tu et al. (2021) proposed a quantum-behaved SSA approach and studied the application of the advocated approach in wireless sensor networks. In, Wang et al. (2021) developed an improved SSA with opposition based learning mechanism and ranking-based learning strategy for global optimization problems and PV parameter extraction task.

Although the SSA algorithm has shown excellent performance on global optimization problems, it still suffers from premature convergence and insufficient solution accuracy when large-scale optimization tasks and complex restricted engineering design issues. To address these limitations of the standard SSA, many high-performance SSA-based algorithms have been developed. In, Ding et al. (2022) developed a velocity-based SSA algorithm. The proposed algorithm enhances the search efficiency of SSA by limiting the maximum speed of the algorithm. Furthermore, an adaptive mechanism was added to the SSA to balance the exploration and exploitation ability. The introduced algorithm was tested using the CEC 2017 benchmark suite. Experimental results show that the velocity-based SSA algorithm outperforms all competitors. Finally, the proposed algorithm is employed to solve the mobile robot path planning task, and the results show that the algorithm is able to plan reasonable collision-free path for the robot. In (Çelik et al. (2021), devised three simple but efficacious strategies to improve the performance of the standard SSA. First, the control parameter is amended chaotically to enhance the tradeoff between exploration and exploitation. Then, a new mutualistic phase is injected to augment the information exchange between leading salps. Finally, stochastic techniques are applied to improve the dynamics among the followers. In, Chen et al. (2022) made three adjustments to the basic SSA. Opposition-based learning technique is adopted to enrich the population diversity. The leader location update formula is modified to help the salp chain jump out of sub-optimal solution. A social learning tactic inspired by PSO is introduced to accelerate the convergence of the optimizer. In, Zhang et al. (2021) introduced a mutual learning mechanism in the exploitation phase of SSA to improve its performance, and used a tangent factor to update the location of the search agent. In, Wang et al. (2022c) designed an orthogonal quasi-opposition-based learning structure to avoid the population from falling into local optima. Moreover, a dynamic learning paradigm is proposed to effectively improve the search pattern of the followers. In (Bairathi and Gopalani, 2021), Bairathi et al. proposed a boosted version of SSA for complex multimodal problems. First, stochastic opposition-based learning is used to enhance the ability to search for unknown regions. Then, multiple search agents are employed to serve as leaders instead of one to intensify the global search ability. Finally, it is compounded with the simulation annealing algorithm to improve the local development ability. In, Singh et al. (2022) proposed a hybrid algorithm of HHO and SSA to cover the unbalanced local search and global exploration of the basic SSA.

Many existing SSA variants focus mainly on alleviating the shortcomings of lack of convergence accuracy and unbalanced exploitation and exploration suffered by the basic SSA. For this purpose, different strategies have been injected into SSA and achieved remarkable results. However, these two limitations have not been completely solved and there are still research gaps. Moreover, the “No Free Lunch” theorems (Wolpert and Macready, 1997) logically proves that it is impossible to expect one algorithm to solve all optimization problems. That is to say, while each algorithm has some unique characteristics along with shortcomings. Even for reputable algorithms, they still have some limitations. For example, AMO is a classical metaheuristic algorithm inspired by animal migration behavior. This optimizer can effectively improve the initial random population and converge to the global optima. It has many advantages, including simple search pattern, easy to implement, and strong global optimization ability. Thanks to the success of the AMO algorithm, it has been applied to many different optimization problems, such as clustering (Hou et al., 2016), the optimal power flow problem (Dash et al., 2022), and multilevel image thresholding (Rahkar Farshi, 2019). However, as most metaheuristic techniques, it suffers from premature convergence and often falls into optima. To solve this drawback, many AMO variants have been proposed, such as the opposition-based AMO (Cao et al., 2013), and Lévy flight assisted AMO (Gülcü,, 2021). Differential evolution (DE) (Storn and Price, 1997) is a global search algorithm, which simulates the biological process in the nature. In DE algorithm, individuals repeatedly perform mutation, crossover, and selection to guide the search process to gradually approach the global optima solution. Because of its simplicity and effectiveness, it has received a lot of attention and has been applied to solve many real-world problems (Wang et al., 2022d). However, DE has some limitations, such as unbalanced exploration and exploitation ability and being sensitive to the selection of the control parameters. To alleviate these drawbacks, Li et al. (2017) proposed a Multi-search DE algorithm with three adjustments. First, the population is divided into multiple subpopulations and the subpopulation group size is dynamically adjusted. Second, three effective mutation strategies are proposed to take on the responsibility for either exploitation or exploration. Finally, a novel parameter adaptation method is designed to solve the automatically adjust the algorithmic parameters. However, there is no mechanism in this DE variant for large-scale problems, which will result in its performance will still be restricted by the phenomenon of “curse of dimensionality” as the number of dimensions increase. The CS is an effective optimization algorithm with two features that make it stands out against other metaheuristic techniques. First, it uses a mutation function based on Lévy flight to improve the quality of the randomly selected solutions at each iteration. Second, it uses one parameter called abandon fraction that does not require fine-tuning. However, CS suffers from the drawback of being prone to premature convergence (Zhang et al., 2019). To address this limitation, many CS variants were developed. For example, Li et al. (Li and Yin, 2015) used two novel mutation rules and the new rules were combined by a linear decreasing probability rule to balance the exploitation and exploration of the algorithm. Further, the parameter setting was adjusted to enhance the diversity of the population. The performance of the developed CS-based method was tested using 16 classical test functions. Experimental results show that the introduced approach performs better than its competitors, or at least comparable to the peer algorithms. However, according to the statistical results, the proposed algorithm still suffers from the drawback of insufficient convergence accuracy. In this paper, a novel SSA variant called OPLSSA is introduced considering the above two considerations. First, a novel pinhole-imaging opposition-based learning mechanism is deigned and combined with the orthogonal experimental design for effectively enhancing the global exploration ability. Second, the follower update pattern is modified by introducing adaptive inertia weights to provide dynamic search with adaptive mechanism. Comprehensive comparison experiments on 23 widely used numerical test functions and 30 CEC 2017 benchmark problems demonstrate that the proposed approach outperforms the traditional SSA, popular SSA-based methods, and well-established population-based intelligent algorithms.

The remainder of this work is structured as follows: The standard SSA, including the principle, mathematical model and shortcoming analysis, is presented in Section 2. The developed modifications and the advocated OPLSSA algorithm are described in detail in Section 3. The effectiveness of the proposed approach is verified by comparison experiments in Section 4. Finally, the conclusions and future tasks are provided in Section 5.

## 2 The original salp swarm algorithm

### 2.1 Description of salp swarm algorithm

The SSA algorithm is a well-established swarm intelligent approach inspired by the unexplained behavior of salp swarm that organize in chains to improve foraging efficiency in oceans. SSA, resembling other population intelligence-based methodologies, commences its search process with a suit of randomly generated search agents, each of which indicates a solution to the pending problem. SSA compartmentalizes the salp population into two groups: leaders and followers. The leader is the key member, which plays a leadership role and at the front of the chain to lead the population in search of food. The followers, on the other hand, move implicitly or outright along the trajectory of the leader.

In SSA, the leading salp changes position depending on the following formula.

$$X_{1,j}=\left\{\begin{array}{l} F_{j}+c_{1}\times \left(\left(ubj-lbj\right)\times c_{2}+lbj\right)\;c_{3}\geq 0.5\\ F_{j}-c_{1}\times \left(\left(ubj-lbj\right)\times c_{2}+lbj\right)\;c_{3}<0.5 \end{array}\right.$$ (1)

where *X*_1,__*j*_ and *F*_*j*_ are the locations of the leader and the food source, respectively, *c*_2_ is a random vector, *c*_3_ is a random value, all taking values between the interval [0,1], and *c*_1_ is the key parameter that regulates the transformation of the algorithm from the exploration in the initial iteration to the exploitation in the later search stages, which is calculated according to the following equation.

$$c_{1}=2\times e^{-{\left(\frac{4\times l}{L}\right)}^{2}}$$ (2)

where *l* and *L* denote the current iteration and the maximum iteration, respectively.

The mathematical equation used to change the followers’ positions is as follows:

$$X_{i,j}=\frac{1}{2}\times \left(X_{i,j}+X_{i-1,j}\right)$$ (3)

where *X*_*i*,*j*_ indicates the location of the *i*-th follower in the *j*th dimensional search landscape.

Algorithm 1 outlines the pseudo code of the basic SSA.

Algorithm 1 *Q*-Pseudocode of SSA.

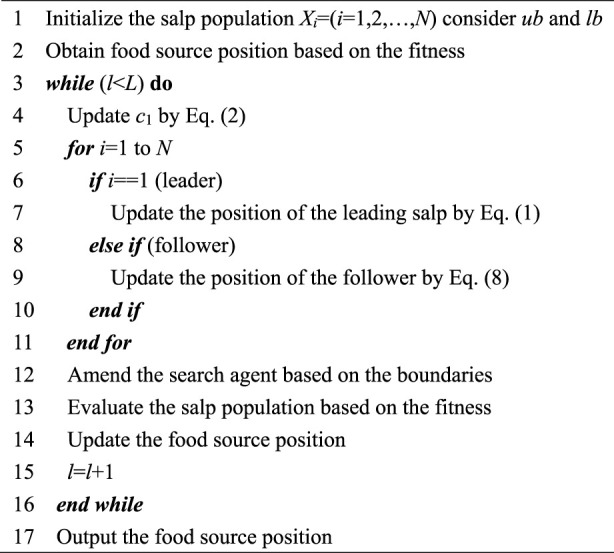

The SSA approach begins the search with a reservation number of randomly generated search agents, and subsequently continuously updates the population position according to the objective value of the optimized function. The fitness of the population is evaluated after each iteration and the best individual is assigned as the current food source, which is the desired goal pursued by the leader, and the followers intuitively or implicitly follow the leading salp, the salp chain thus continuously approaching the food source. Notably, the value of the control parameter *c*_1_ decreases nonlinearly after the lapse of iterations, and the salp chain accordingly switches from moving in large steps to explore the search space to traveling gradually to exploit the already discovered potential areas. The salp swarm repeatedly searches following the aforementioned pattern until the cessation criterion is encountered, at which point the food source is the expected optimal solution.

### 2.2 Analysis of the shortcomings of salp swarm algorithm

In this subsection, we analyze the key limitations of the basic SSA, which is the inference and motivation behind the current work. The details are as follows:

1) First of all, there is only one parameter *c*_1_ to be updated in the basic SSA, which is used to control the movement of the leader and thus maintain a more stable balance between exploration and exploitation. However, strong stochastic factors break this expectation. The food source could not guide the leader salp to the more promising search region as expected, resulting in the algorithm not being able to switch search modes smoothly during the search process, which also reduced the convergence speed.
2) Second, the position update equation of the followers in SSA does not have any control parameters, and although this can reduce the computational cost, it will make this movement mechanism sluggish and the algorithm is thus prone to fall into local optimum.
3) In addition, adaptive is a novel and effective technique that helps the algorithm to adjust the movement pattern autonomously during the search process, however, such operator is lacking in SSA.
4) Finally, maintaining a desirable balance between exploitation and exploration is the goal pursued by all swarm-based intelligence techniques, and a lot of research has focused on enhancing the capability of SSA algorithms in this regard to strengthen its overall performance, but there is still a gap in this context.

The above analyzed weak points of SSA have promoted the authors to discern that the algorithm has some drawbacks to be rectified, and this is the motivation behind proposing a novel version of SSA. Each of the embedded adjustments will be described in detail in the next section.

## 3 Essentials of the OPLSSA

As discussed previously, the swarm intelligent algorithm covers two phases, namely exploration and exploitation. Exploration maintains a superiority in global search, and the strong exploration capability is conductive to improving the convergence speed. On the other hand, the domain nature of exploitation is shown in the local search, and the powerful development capability is beneficial to boost the convergence accuracy. Maintaining a proper equilibrium between exploration and exploitation holds a key position in the performance of swarm intelligent approaches, which is also a research gap that the current community tries to bridge. In this study, two straightforward but applicable mechanisms are integrated into SSA to produce an enhanced balanced SSA variants with better performance. This section provides an in-depth discussion of the designed components and the OPLSSA algorithm.

### 3.1 Orthogonal pinhole-imaging-based learning

In standard SSA, according to the location changing pattern of the leading salp, a prospective candidate search agent location is gained by directing the leader to the food source. Followers chase the leader explicitly or indirectly, gathering near the perceived global optimum in the later phase of the search. As a result, the standard SSA is inclined to converge prematurely. Therefore, improving the ability of the approach to avoid local optima has been considered as the most critical and necessary research goal in SSA improvement. To enhance the global exploration ability of swarm intelligent metaheuristic techniques, the most common method used in the published literatures is opposition-based learning (OBL). For example, Abualigah et al. (2021) revised the search pattern of the slime mould algorithm (SMA) (Li et al., 2020) by incorporating the OBL mechanism. Dinkar et al. (2021) used the OBL technique to accelerate the convergence rate of equilibrium optimizer (EO) (Faramarzi et al., 2020b), and the reported OBL-based EO approach was used for multilevel threshold image segmentation. Yu et al. (2021) included an additional OBL phase in the GWO algorithm to help the wolves jump to reverse individuals to enhance the exploration of the search space. Yildiz et al. (2021) mixed OBL with GOA algorithm to improve the ability of the algorithm to exploit unknown regions. In the proposed algorithm, elite individuals generate corresponding elite reverse individuals through the OBL strategy and retain well-quality individuals for the next iteration. Chen et al. (2020) employed quasi-opposition-based learning (QOBL), an OBL variant, to create dynamic jumps during the location update of the WOA algorithm to prevent the algorithm from falling into local optima.

**FIGURE 1:**
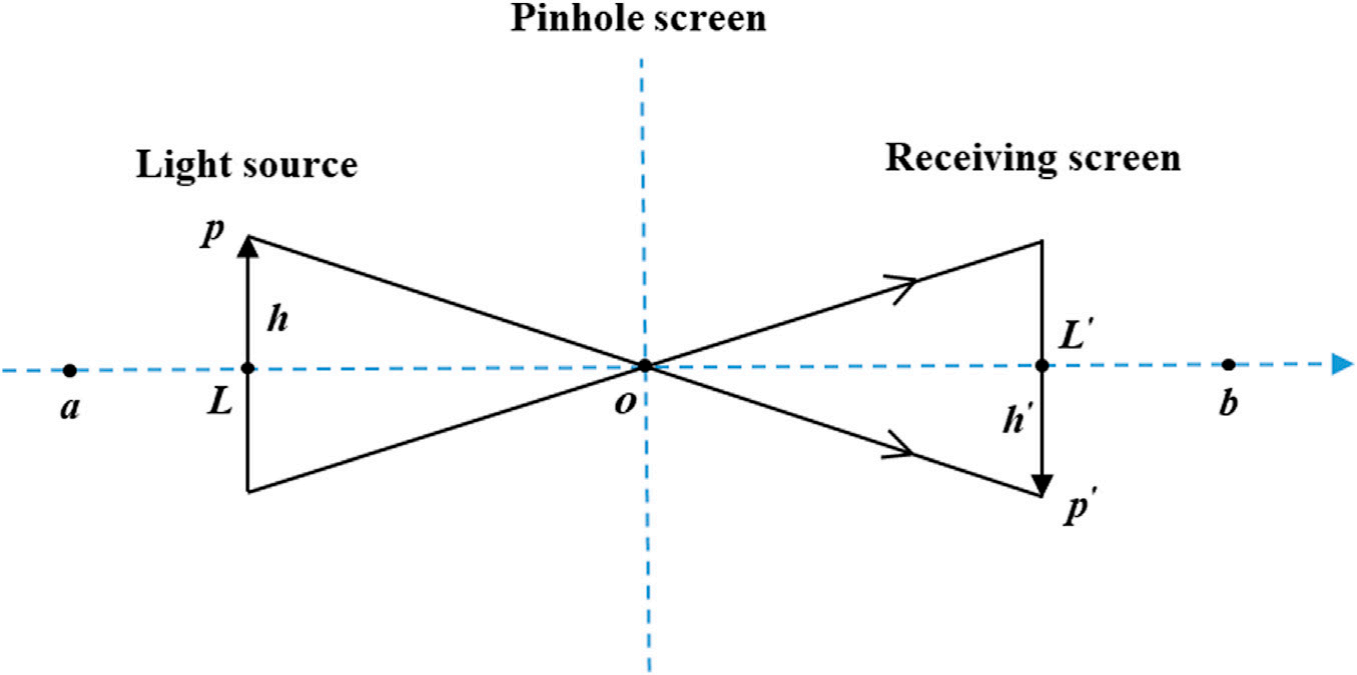
The schematic diagram of PIBL on the leading salp.

Pinhole imaging is a general physical phenomenon in which a light source passes through a small hole in a plate and an inverted real image is formed on the other side of the plate. Motivated by the discovery that there are close similarities between the pinhole imaging phenomenon and the OBL mechanism, this paper proposes a pinhole-imaging-based learning (PIBL) mechanism and applies it to the current leader to augment the exploration capability of SSA for unknown areas. Figure 1 plots the schematic diagram of the PIBL.

In Figure 1, *p* is a light source of height *h*, and its projection on the *x*-axis is *L*. Place a pinhole screen on the base point *o*, and the real image *p*′ formed by the light source through the pinhole screen will fall on the receiving screen on the other side. It is worth noting that *p*′ is the inverted image of *p*, and the height of *p*′ from the x-axis is *h*′. At this point, *L* jumps to *L*′ based on pinhole imaging principle. Therefore, from the pinhole imaging principle, it can be derived that

$$\frac{\left(a+b\right)/2-L}{L^{\prime }-\left(a+b\right)/2}=\frac{h}{h^{\prime }}$$ (4)

Let *h*/*h’* = *n*, Eq. 9 can be rewritten as

$$L^{\prime }=\frac{\left(a+b\right)}{2}+\frac{\left(a+b\right)}{2n}-\frac{L}{n}$$ (5)

Equation 5 is the formula of PIBL on the leading salp. When *n* = 1, the above equation is simplified to

$$L^{\prime }=a+b-L$$ (6)

Equation 6 is the original OBL strategy on the leading salp. Clearly, OBL is very similar to PIBL. In other words, PIBL can be treated as a dynamic version of OBL.

Generalizing Eq. 5 to the *D*-dimensional spatial, it can be obtained

$$L_{j}^{\prime }=\left(a_{j}+b_{j}\right)/2+\left(a_{j}+b_{j}\right)/2k-L_{j}/n$$ (7)

where *L*_*j*_ and *L*′ *j* are the *j*th dimensional values of the leader and the PIBL leader, respectively.

In this paper, we use the proposed PIBL mechanism to help the leader search for unknown regions, thus improving the global search ability of the algorithm and avoiding the premature convergence due to the lack of exploration capability. However, similar to OBL, PIBL also suffers from the problem of “dimensional degradation”, i. t., the current leader improves only in some dimensions after jumping to the PIBL leader, while some other dimensions are even farther from the global optimum. To solve this problem, we introduce orthogonal experimental design (OED) and combine it with the PIBL strategy to design the orthogonal pinhole-imaging-based learning (OPIBL) mechanism.

The OED is an auxiliary tool that can find the optimal combination of experiments by a reasonable number of trials. For example, for an experiment with 3 levels and 4 factors, it would take 81 attempts using a trial-and-error approach. In contrast, by adopting an OED, only 9 sets of representative combinations need to be evaluated to determine the optimal combination of the experiment. *L*_9_ (3^4^) is shown in Table 1.

**TABLE 1:** Orthogonal array of *L*_9_ (3^4^).

*M*	*K*			
	1	2	3	4
	1	1	1	1
2	1	2	2	2
3	1	3	3	3
4	2	1	2	3
5	2	2	3	1
6	2	3	1	2
7	3	1	3	2
8	3	2	1	3
9	3	3	2	1

**FIGURE 2:**
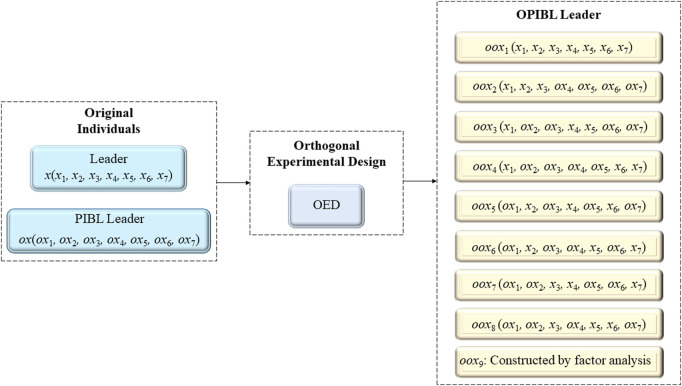
Construct OPIBL leader.

In each iteration, the OPIBL mechanism is used for the leader, the dimension of the problem to be solved is considered as the factor of the OED, and the leader and the PIBL leader are regarded as the two levels of the OED. The designed OPIBL mechanism considers the information of the current leader and the PIBL leader, and retains the respective dominant dimensions to combine as a promising partial PIBL individual, called OPIBL leader. In this way, the OPIBL mechanism can effectively avoid the “dimensional degradation” problem caused by PIBL and significantly help the leader to quickly approach the global optimal solution. To visualize the process of the leader jumps to OPIBL leader, we consider a 7-dimensional problem and draw the schematic diagram of the leader jumps to the OPIBL leader according to *L*_8_ (2^7^), as shown in Figure 2.

In order not to increase the computational complexity of the algorithm, only the leader executes the OPIBL operation. Then, evaluate both the current leader and the OPIBL leader, and reserve the high-quality search agent.

### 3.2 Adaptive conversion parameter strategy

For nature-inspired swarm intelligent algorithms, strong exploration capability is beneficial to improve the convergence speed, while powerful exploitation ability is conductive to refine the convergence accuracy. Maintaining a proper balance between exploration and exploitation can effectively boost the overall performance of the algorithm, which is a research difficulty that the metaheuristic community has been trying to conquer with great effort. In the basic SSA, the follower updates its position according to Eq. 3. This equation is control parameter-free, the current follower salp only considers its own position and the position of neighboring individual to calculate the next location. Although this mechanism makes SSA more consistent with the advocated minimalism, this rigid position update pattern tends to deviate the population from the global optimum. Furthermore, it is unreasonable that the followers move without utilizing the current global optimal position. To address this issue, many studies have focused on modifying the follower position update equation to enhance the dynamic nature.

Based on the above analysis, this paper proposes an adaptive position update mechanism for follower salps to replace the original formula, namely

$$X_{i,j}=\frac{1}{2}\times \left(\omega X_{i,j}+X_{i-1,j}\right)$$ (8)

where *ω* is an inertia weight factor.

In PSO, the inertia weight coefficient factor changes dynamically during the search process to help the algorithm switch between exploitation and exploration operations. With the progress of the research on metaheuristic techniques, the inertia weight has been introduced into many swarm intelligence-based approaches to improve their performance. For example, GU et al. (2022) implemented the inertia weight to tune the particle’s search behavior in chicken swarm algorithm (CSA) (Meng et al., et al.). Jena et al. (Jena and Satapathy, 2021) used a Sigmoid adaptive inertia weight to intensify the performance of the social group optimization (SGO) (Naik et al., 2018). Inspired by the above studies, a novel inertia weight coefficient is proposed in this work with the following mathematical expression:

$$\omega =\left(\omega_{\max}-\omega_{\min}\right)\cdot \frac{e^{10-\lambda t}-2}{e^{10-\lambda t}+2}+\omega_{\max}$$ (9)

where *λ* is a constant number, *ω*_max_ and *ω*_min_ are the maximum and minimum values of the inertia weight coefficient, respectively.

**FIGURE 3:**
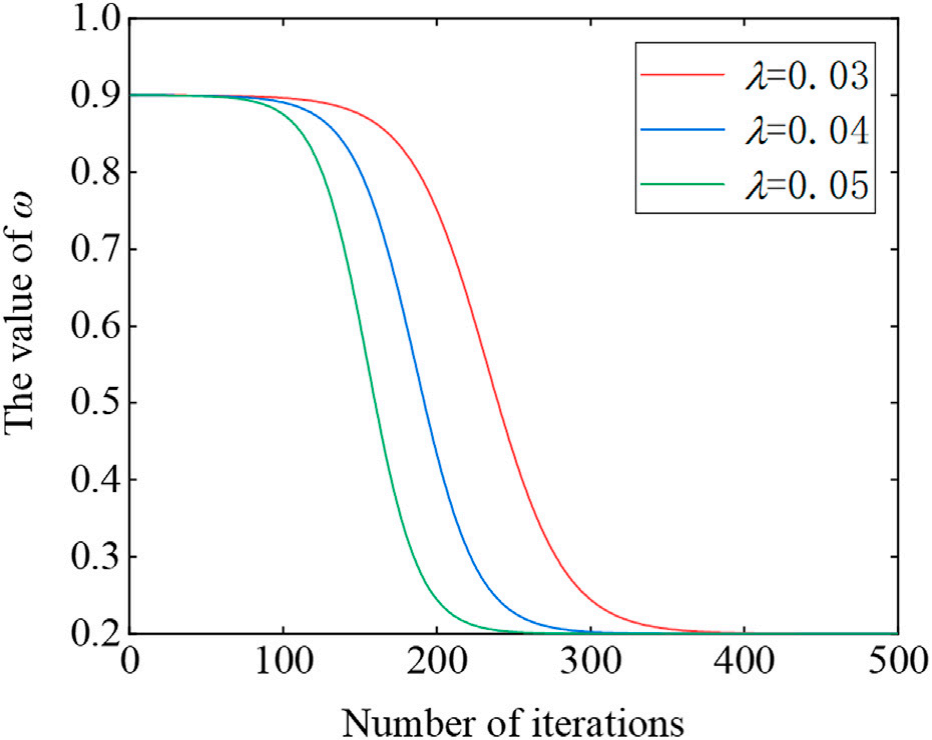
The inertia weight *ω* curve.

Figure 3 plots the schematic diagram of the nonlinear decrease of the inertia weight during the iterative process. From the figure, in the initial stage, the value of *ω* is larger, and the particle accordingly moves in larger steps in the search space, which is beneficial to the global search. After the lapse of iterations, *ω* nonlinearly decreases and the particle moves in shorter steps correspondingly, which is advantageous for fine exploit the already explored promising area to improve the convergence accuracy.

### 3.3 The flowchart of OPLSSA

**FIGURE 4:**
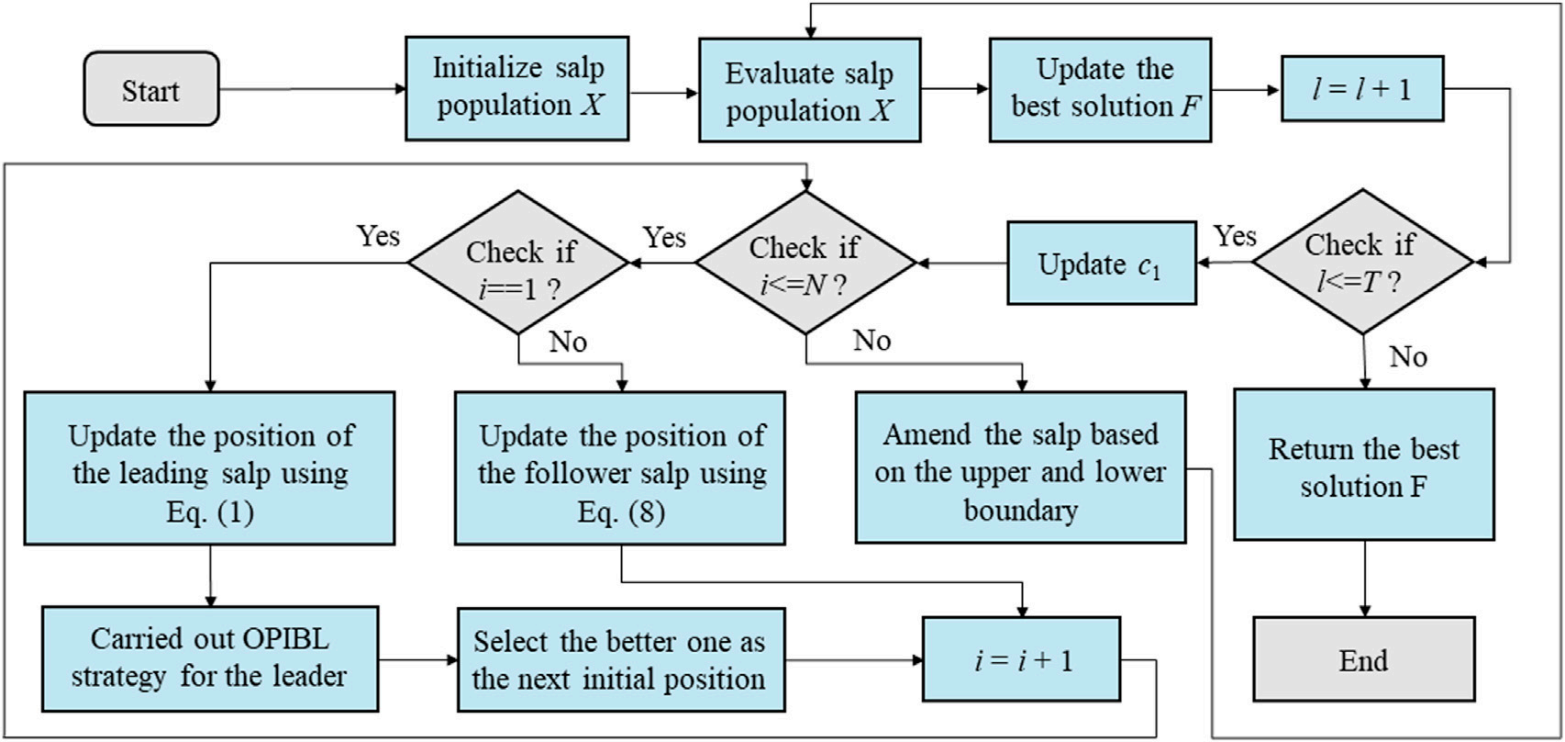
The flow chart of OPLSSA.

In summary, Figure 4 shows the flow chart of the developed OPLSSA algorithm.

## 4 Simulations and comparisons

In this section, 23 classical benchmark test functions are solved using the OPLSSA algorithm to synthetically verify its effectiveness and applicability. The results obtained by the proposed approach on the test cases are recorded and compared with some well-established metaheuristic techniques, including the basic SSA, the forefront swarm intelligent algorithms, and the popular SSA variants. All experiments were implemented under MATLAB 2016b software, operating system used is Microsoft WINDOWS 10 64-bit Home, and simulations supported by Intel (R) Core (TM) i7-7700 CPU at 3.60 GHz with 8.00 GB RAM.

### 4.1 Experiments on well-known benchmark functions

In this subsection, the selected 23 benchmark functions that include both multimodal and unimodal functions are reported, as shown in Table 2, where Search range represents the boundary of the function search space, and *f*_min_ is the best value. Among them, *f*_1_-*f*_11_ are unimodal functions, and they are mainly complex spherical or valley value problems. They have only one global optimal solution in the search range but are difficult to find. Therefore, it can be used to test the convergence efficiency and exploration ability of each algorithm. Different from the unimodal functions, the multimodal functions (*f*_12_-*f*_23_) have multiple local extremes in the search space. Moreover, the scale of this type of problem will increase exponentially as the dimensionality increases. Therefore, the multimodal problem can effectively test the abilities of each algorithm to search globally and to jump out of local optima (Long et al., 2021).

**TABLE 2:** The characteristics of the classical benchmark functions.

Function type	Function formulation	Search range	*f*_min_
Unimodal	$f_{1}\left(x\right)=\sum_{i=1}^{D}x_{i}^{2}$	[−100,100]	0
	$f_{2}\left(x\right)=\sum_{i=1}^{D}ix_{i}^{2}$	[−10,10]	0
	$f_{3}\left(x\right)=\sum_{i=1}^{D}{\left(\sum_{j=1}^{i}x_{j}\right)}^{2}$	[−100,100]	0
	$f_{4}\left(x\right)=\max_{i}\left\{\left|x_{i}\right|,1\leq x_{i}\leq D\right\}$	[−100,100]	0
	$f_{5}\left(x\right)=\sum_{i=1}^{D}{\left(\left\lfloor x_{i}+0.5\right\rfloor \right)}^{2}$	[−100,100]	0
	$f_{6}\left(x\right)=\sum_{i=1}^{D}ix_{i}^{4}$	[−1.28,1.28]	0
	$\left.f_{7}\left(x\right)=\sum_{i=1}^{D}ix_{i}^{4}+random[0,1\right)$	[−1.28,1.28]	0
	$f_{8}\left(x\right)=\sum_{i=1}^{D}{\left|x_{i}\right|}^{\left(i+1\right)}$	[−1,1]	0
	$f_{9}\left(x\right)=\sum_{i=1}^{D}{\left(10^{6}\right)}^{\left(i-1\right)/\left(D-1\right)}x_{i}^{2}$	[−100,100]	0
	$f_{10}\left(x\right)=x_{1}^{2}+10^{6}\cdot \sum_{i=2}^{D}x_{i}^{6}$	[−100,100]	0
	$f_{11}\left(x\right)=10\cdot x_{1}^{2}+\sum_{i=2}^{D}x_{i}^{6}$	[−1,1]	0
Multimodal	$f_{12}\left(x\right)=\sum_{i=1}^{D}\left[x_{i}^{2}-10\cos\left(2\pi x_{i}\right)+10\right]$	[−5.12,5.12]	0
	$f_{13}\left(x\right)=-20\exp\left(-0.2\sqrt{\frac{1}{D}\sum_{i=1}^{D}x_{i}^{2}}\right)-\exp\left(\frac{1}{D}\sum_{i=1}^{D}\cos\left(2\pi x_{i}\right)\right)+20+e$	[−32,32]	0
	$f_{14}\left(x\right)=\frac{1}{4000}\sum_{i=1}^{D}x_{i}^{2}-\prod_{i=1}^{D}\cos\left(\frac{x_{i}}{\sqrt{i}}\right)+1$	[−600,600]	0
	$f_{15}\left(x\right)=\sum_{i=1}^{D}\left|x_{i}\cdot \sin\left(x_{i}\right)+0.1x_{i}\right|$	[−10,10]	0
	$f_{16}\left(x\right)={\sin\;}^{2}\left(\pi x_{1}\right)+\sum_{i=1}^{D-1}\left[x_{i}^{2}\cdot \left(1+10{\sin\;}^{2}\left(\pi x_{1}\right)\right)+{\left(x_{i}-1\right)}^{2}-{\sin\;}^{2}\left(2\pi x_{i}\right)\right]$	[−10,10]	0
	$f_{17}\left(x\right)=0.1D-\left(0.1\sum_{i=1}^{D}\cos\left(5\pi x_{i}\right)-\sum_{i=1}^{D}x_{i}^{2}\right)$	[−1,1]	0
	$f_{18}\left(x\right)=\sum_{i=1}^{D}x_{i}^{2}+{\left(\sum_{i=1}^{D}0.5x_{i}\right)}^{2}+{\left(\sum_{i=1}^{D}0.5x_{i}\right)}^{4}$	[−-5,10]	0
	$f_{19}\left(x\right)=\sum_{i=1}^{D}\left(0.2x_{i}^{2}+0.1x_{i}^{2}\cdot \sin\left(2x_{i}\right)\right)$	[−10,10]	0
	$f_{20}\left(x\right)={\left[\frac{1}{D-1}\sum_{i=1}^{D}\left(\sqrt{x_{i}}\left(\sin\left(50.0x_{i}^{0.2}\right)+1\right)\right)\right]}^{2}$	[−100,100]	0
	$f_{21}\left(x\right)=\sum_{i=1}^{D-1}\left[x_{i}^{2}+2x_{i+1}^{2}-0.3\cos\left(3\pi x_{i}\right)-0.4\cos\left(4\pi x_{i+1}\right)+0.7\right]$	[−15,15]	0
	$f_{21}\left(x\right)=\sum_{i=1}^{D-1}\left.{\left(x_{i}^{2}+2x_{i+1}^{2}\right)}^{0.25}\cdot {\left((\sin50(x_{i}^{2}+x_{i+1}^{2}\right)}^{0.1}{)}^{2}+1\right)$	[−10,10]	0
	$f_{23}\left(x\right)=\sum_{i=1}^{D-1}x_{i}^{6}\cdot \left(2+\sin\frac{1}{x_{i}}\right)$	[−1,1]	0

#### 4.1.1 Compared against salp swarm algorithm and salp swarm algorithm variants

To test the performance of the advocated OPLSSA algorithm, 23 classical benchmark functions reported in Table 2 were employed. The dimensions of the involved problems were set to 100. The obtained results were compared with the standard SSA and seven representative SSA variants, including the self-adaptive SSA (ASSA) (Salgotra et al., 2021), adaptive SSA with random replacement strategy (RDSSA) (Ren et al., 2021), lifetime tactic enhanced SSA (LSSA) (Braik et al., 2020), the Gaussian perturbed SSA (GSSA) (Nautiyal et al., 2021), the intensified OBL-based SSA.

(OBSSA) (Hussien, 2021), inertia weight enhanced SSA (ASSO) (Ozbay and Alatas, 2021), and WOA improved SSA (IWOSSA) (Saafan and El-Gendy, 2021). The general parameters of the involved methods were set as recommended in the respective original literature. In OPLSSA, *k* = 100, *λ* = 0.04, *ω*_max_ = 0.55, *ω*_min_ = 0.2. The maximum number of iterations of each algorithm for each benchmark was 500, the population size was set to 30, and the number of independent runs was set to 30 to eliminate random errors. The mean value and standard variance of the results obtained from 30 runs were recorded as metrics to evaluate the performance of the algorithms. In addition, Friedman test was utilized to evaluate the average performance of the algorithms. Wilcoxon signed ranks was used as an auxiliary tool to investigate the differences between OPLSSA and its competitors from a statistical point of view. The results of the nine algorithms on the 23 tested problems are shown in Table 3, and the results of the Wilcoxon signed ranks test are reported in Table 4.

**TABLE 3:** Comparisons of nine algorithms on 23 test functions with 100 dimensions.

Function	Results	SSA	LSSA	ASSA	GSSA	OBSSA	ASSO	RDSSA	IWOSSA	OPLSSA
f1	Mean	1.29E+03	2.50E-03	5.11E-02	1.23E-15	3.92E-32	2.02E-26	3.93E-65	1.75E-06	0
	Std	3.17E+02	2.20E-03	3.97E-02	4.22E-15	4.65E-32	3.11E-27	1.75E-64	1.61E-06	0
	f-rank	9	7	8	5	3	4	2	6	1
f2	Mean	8.71E+02	2.00E-01	2.01E-02	7.11E-16	3.00E-32	9.87E-27	8.16E-39	4.69E-07	0
	Std	2.42E+02	1.74E-01	8.20E-03	2.86E-15	3.50E-32	1.48E-27	4.47E-38	4.08E-07	0
	f-rank	9	8	7	5	3	4	2	6	1
f3	Mean	5.02E+04	8.39E+03	1.79E+04	1.43E+04	4.77E-30	1.92E-25	5.28E-34	1.01E+05	0
	Std	2.29E+04	5.89E+03	9.86E+03	8.51E+03	8.26E-30	7.04E-26	2.89E-33	3.21E+04	0
	f-rank	8	5	7	6	3	4	2	9	1
f4	Mean	2.73E+01	2.25E+01	9.3313	2.39E+01	5.52E-17	3.45E-14	5.53E-30	4.47E+01	0
	Std	2.8509	5.2878	2.6632	3.2136	3.56E-17	3.08E-15	2.40E-29	7.0144	0
	f-rank	8	6	5	7	3	4	2	9	1
f5	Mean	2.83E+03	8.1000	1.46E+01	0	0	0	0	1.0667	0
	Std	8.76E+02	5.0402	7.0392	0	0	0	0	1.7991	0
	f-rank	9	7	8	1	1	1	1	6	1
f6	Mean	2.97E-01	1.17E-02	1.06E-07	1.86E-02	3.97E-70	1.44E-59	1.95E-95	3.67E-12	0
	Std	1.91E-01	1.70E-02	1.14E-07	2.55E-02	7.31E-70	4.82E-60	1.07E-94	8.94E-12	0
	f-rank	9	7	6	8	3	4	2	5	1
f7	Mean	2.6326	6.81E-01	8.53E-02	1.13E-01	7.85E-05	1.14E-04	7.41E-04	8.06E-02	8.96E-05
	Std	4.78E-01	2.71E-01	2.09E-02	1.45E-01	9.21E-05	1.03E-04	5.22E-04	4.73E-02	9.53E-05
	f-rank	9	8	6	7	1	3	4	5	2
f8	Mean	3.36E-06	2.59E-10	1.98E-26	7.00E-50	9.10E-39	1.17E-35	2.74E-74	1.33E-17	0
	Std	2.60E-06	1.21E-09	7.55E-26	3.83E-49	2.83E-38	2.33E-35	1.50E-73	4.51E-17	0
	f-rank	9	8	6	3	4	5	2	7	1
f9	Mean	7.14E+07	1.2397	1.26E+02	1.20E-14	2.88E-27	1.36E-21	6.63E-33	9.80E-03	0
	Std	2.67E+07	9.42E-01	5.88E+01	4.08E-14	4.05E-27	5.33E-22	3.63E-32	6.50E-03	0
	f-rank	9	7	8	5	3	4	2	6	1
f10	Mean	6.72E+13	1.36E+09	9.78E+05	1.53E+13	5.34E-35	5.37E-32	2.55E-82	1.69E+05	0
	Std	4.72E+13	3.58E+09	1.12E+06	1.27E+13	1.55E-34	7.22E-32	1.39E-81	5.07E+05	0
	f-rank	9	7	6	8	3	4	2	5	1
f11	Mean	1.7951	1.47E-04	2.78E-10	5.71E-17	7.60E-33	5.19E-30	5.47E-76	5.77E-09	0
	Std	1.5770	3.60E-04	7.29E-10	2.49E-16	2.92E-32	1.15E-29	2.99E-75	2.62E-08	0
	f-rank	9	8	6	5	3	4	2	7	1
f12	Mean	2.36E+02	1.95E+02	2.38E+02	2.88E-11	0	0	0	3.22E+02	0
	Std	3.42E+01	1.13E+02	1.31E+02	1.27E-10	0	0	0	1.14E+02	0
	f-rank	7	6	8	5	1	1	1	9	1
f13	Mean	1.03E+01	8.47E-02	2.93E-02	1.32E-09	8.88E-16	1.96E-14	4.20E-15	6.63E-01	8.88E-16
	Std	1.4847	3.05E-01	1.03E-02	1.80E-09	0	2.27E-15	9.01E-16	3.6301	0
	f-rank	9	7	6	5	1	4	3	8	1
f14	Mean	1.35E+01	2.74E-02	5.33E-02	1.29E-14	0	0	0	3.60E-03	0
	Std	4.7887	4.76E-02	3.61E-02	6.18E-14	0	0	0	8.60E-03	0
	f-rank	9	7	8	5	1	1	1	6	1
f15	Mean	2.86E+01	1.35E+01	1.04E+01	4.42E-14	1.49E-17	1.14E-14	1.65E-26	1.88E+01	0
	Std	6.1418	1.29E+01	6.2120	1.40E-13	5.88E-18	7.75E-16	6.69E-26	1.56E+01	0
	f-rank	9	7	6	5	3	4	2	8	1
f16	Mean	3.51E+02	1.77E+02	4.31E+01	4.73E-08	1.99E-32	7.97E-27	6.69E-47	8.3098	0
	Std	6.68E+02	1.38E+02	2.49E+01	1.69E-07	1.96E-32	1.22E-27	3.63E-46	2.21E+01	0
	f-rank	9	8	7	5	3	4	2	6	1
f17	Mean	4.9384	1.3822	1.36E-04	0	0	0	0	1.99E-09	0
	Std	6.59E-01	7.74E-01	1.69E-04	0	0	0	0	9.33E-10	0
	f-rank	9	8	7	1	1	1	1	6	1
f18	Mean	7.16E+01	1.78E-02	8.50E-03	9.29E-09	2.09E+02	1.75E-28	4.35E-47	2.16E-02	2.92E-44
	Std	1.95E+01	1.08E-02	4.90E-03	2.12E-08	3.03E+01	3.42E-29	2.38E-46	2.77E-02	8.67E-45
	f-rank	8	6	5	4	9	3	1	7	2
f19	Mean	1.89E+01	3.40E-03	1.48E-04	4.36E-17	8.03E-35	4.02E-29	1.48E-43	2.55E-09	0
	Std	5.7632	4.70E-03	7.83E-05	1.00E-16	9.15E-35	5.04E-30	8.13E-43	1.80E-09	0
	f-rank	9	8	7	5	3	4	2	6	1
f20	Mean	5.0612	2.83E-01	1.8212	5.69E-10	4.79E-09	1.28E-07	6.21E-15	7.26E-02	0
	Std	2.18E-01	238E-01	5.49E-01	5.11E-10	1.36E-09	4.05E-09	2.58E-14	2.26E-01	0
	f-rank	9	7	8	5	4	3	2	6	1
f21	Mean	1.90E+02	1.34E+01	3.94E-01	1.22E-16	0	0	0	1.34E-06	0
	Std	2.88E+01	6.4554	5.12E-01	3.94E-16	0	0	0	7.37E-07	0
	f-rank	9	8	7	5	1	1	1	6	1
f22	Mean	4.1930	4.1724	4.1941	9.39E-01	4.65E-09	1.19E-07	1.86E-02	3.5709	0
	Std	1.88E-01	3.04E-01	1.61E-01	4.69E-01	1.36E-09	4.29E-09	2.18E-02	1.1627	0
	f-rank	8	7	9	5	2	3	4	6	1
f23	Mean	2.59E-04	1.16E-05	5.96E-11	8.13E-05	5.24E-108	1.64E-92	6.43E-209	2.79E-10	0
	Std	1.65E-04	9.04E-06	9.92E-11	1.08E-04	1.72E-107	8.86E-93	0	7.19E-10	0
	f-rank	9	7	5	8	3	4	2	6	1
	Average f-rank	8.7391	7.1304	6.7826	5.1739	2.6956	3.2174	1.9565	6.5652	1.0869
	Overall f-rank	9	8	7	5	3	4	2	6	1

**TABLE 4:** Statistical conclusions based on Wilcoxon signed-rank test on 100-dimensional benchmark problems.

Function	SSA *p*-value	LSSA *p*-value	ASSA *p*-value	GSSA *p*-value	OBSSA *p*-value	ASSO *p*-value	RDSSA *p*-value	IWOSSA *p*-value
f1	1.2118E-12	1.2118E-12	1.2118E-12	1.2118E-12	1.2118E-12	1.2118E-12	1.2118E-12	1.2118E-12
f2	1.2118E-12	1.2118E-12	1.2118E-12	1.2118E-12	1.2118E-12	1.2118E-12	1.2118E-12	1.2118E-12
f3	1.2118E-12	1.2118E-12	1.2118E-12	1.2118E-12	1.2118E-12	1.2118E-12	1.2118E-12	1.2118E-12
f4	1.2118E-12	1.2118E-12	1.2118E-12	1.2118E-12	1.2118E-12	1.2118E-12	1.2118E-12	1.2118E-12
f5	1.2118E-12	1.1808E-12	1.1941E-12	N/A	N/A	N/A	N/A	1.4306E-04
f6	1.2118E-12	1.2118E-12	1.2118E-12	1.2118E-12	1.2118E-12	1.2118E-12	1.2118E-12	1.2118E-12
f7	3.0199E-11	3.0199E-11	3.0199E-11	3.0199E-11	0.6952	0.2062	1.2870E-09	3.0199E-11
f8	1.2118E-12	1.2118E-12	1.2118E-12	1.2118E-12	1.2118E-12	1.2118E-12	4.5736E-12	1.2118E-12
f9	1.2118E-12	1.2118E-12	1.2118E-12	1.2118E-12	1.2118E-12	1.2118E-12	1.2118E-12	1.2118E-12
f10	1.2118E-12	1.2118E-12	1.2118E-12	1.2118E-12	1.2118E-12	1.2118E-12	1.2118E-12	1.2118E-12
f11	1.2118E-12	1.2118E-12	1.2118E-12	1.2118E-12	1.2118E-12	1.2118E-12	1.2118E-12	1.2118E-12
f12	1.2118E-12	1.2118E-12	1.2118E-12	1.4545E-04	N/A	N/A	N/A	1.2118E-12
f13	1.2118E-12	1.2118E-12	1.2118E-12	1.2118E-12	N/A	5.6687E-13	7.1518E-13	1.2118E-12
f14	1.2118E-12	1.2118E-12	1.2118E-12	6.6067E-05	N/A	N/A	N/A	1.2118E-12
f15	1.2118E-12	1.2118E-12	1.2118E-12	1.2118E-12	1.2118E-12	1.2118E-12	1.2118E-12	1.2118E-12
f16	1.2118E-12	1.2118E-12	1.2118E-12	1.2118E-12	1.2118E-12	1.2118E-12	1.2118E-12	1.2118E-12
f17	1.2118E-12	1.2118E-12	1.2118E-12	N/A	N/A	N/A	N/A	1.2118E-12
f18	3.0199E-11	3.0199E-11	3.0199E-11	3.0199E-11	3.0199E-11	3.0199E-11	3.0199E-11	3.0199E-11
f19	1.2118E-12	1.2118E-12	1.2118E-12	1.2118E-12	1.2118E-12	1.2118E-12	1.2118E-12	1.2118E-12
f20	1.2118E-12	1.2118E-12	1.2118E-12	1.2118E-12	1.2118E-12	1.2118E-12	1.2118E-12	1.2118E-12
f21	1.2118E-12	1.2118E-12	1.2118E-12	8.1523E-02	N/A	N/A	N/A	1.2118E-12
f22	8.6253E-13	1.2118E-12	1.2118E-12	1.2118E-12	1.2118E-12	1.2118E-12	1.2078E-12	1.2098E-12
f23	1.2118E-12	1.2118E-12	1.2118E-12	1.2118E-12	1.2118E-12	1.2118E-12	1.2118E-12	1.2118E-12
+/ = /-	23/0/0	23/0/0	23/0/0	21/2/0	17/6/0	17/6/0	17/5/1	23/0/0

From Table 3, the developed OPLSSA is superior to SSA, LSSA, ASSA and IWOSSA on all test functions. Compared to GSSA, OPLSSA presents similar and better performance on two and 21 test cases, respectively. OPLSSA plays a tie with OBSSA on six benchmarks, it beats OBSSA on six functions, and on another function (i.e. *f*_7_), it is inferior to OBSSA. OPLSSAS defeats ASSO on 18 benchmarks, and on the remaining five problems, both algorithms find the theoretical optimal solution. With respect to RDSSA, OPLSSA wins on 17 test functions, ties with it on five test functions, and is defeated by RDSSA on one (i.e. *f*_18_) test function. According to the Friedman ranking achieved by the different approaches for 23 test problems, OPLSSA obtained the top rank, followed by RDSSA, OBSSA, ASSO, GSSA, IWOSSA, ASSA, LSSA and SSA. Furthermore, according to the comparison results of Wilcoxon signed ranks approach, the *p*-values are less than 0.05 except for two pairwise comparisons (i.e. OPLSSA versus OBSSA, OPLSSA versus ASSO), which demonstrates that OPLSSA has a significant advantage over the comparison algorithms.

**FIGURE 5:**
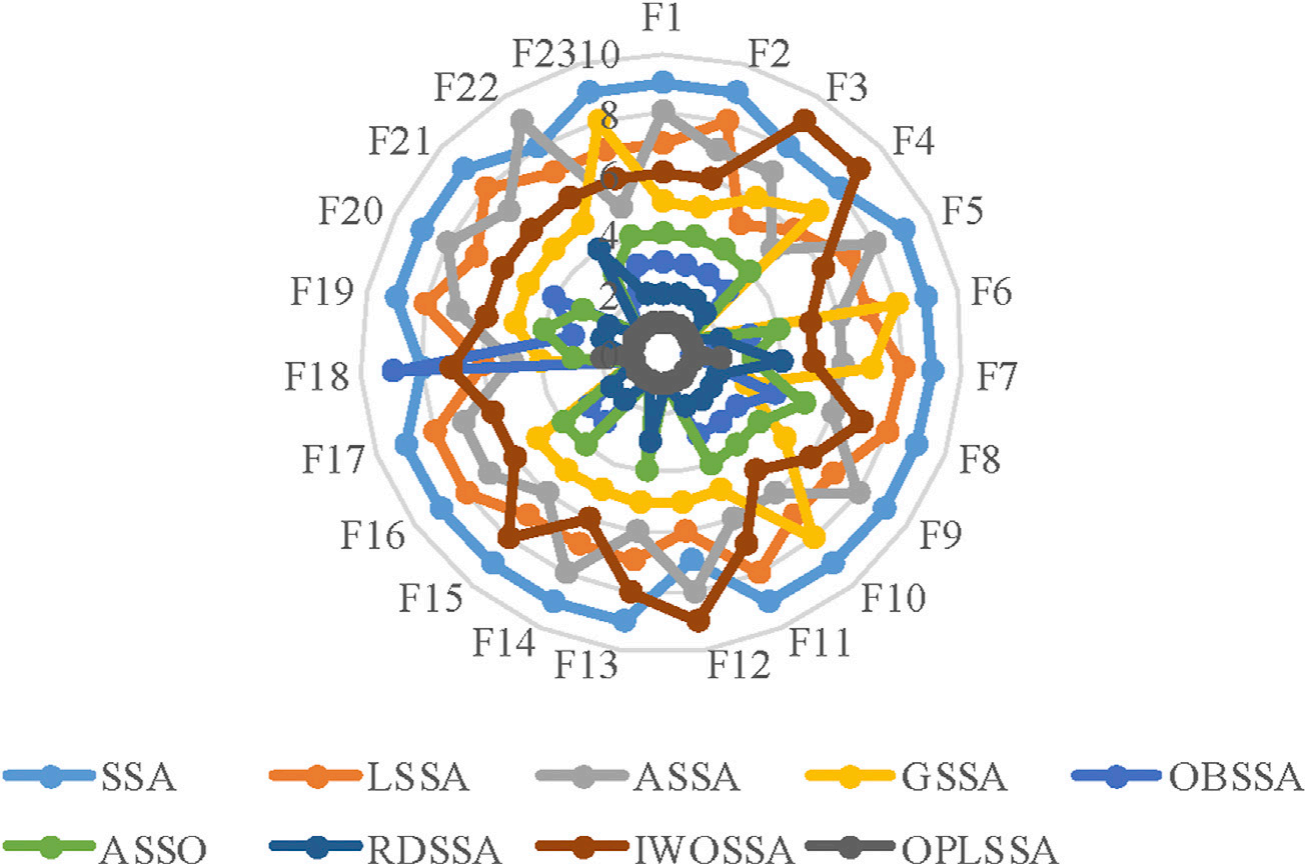
Radar plot for consolidated ranks of 23 benchmarkproblems with the SSA variants.

Figure 5 plots the radar diagram showing the ranking of the nine SSA-based algorithms on the 23 tested functions as counted in Table 4. From the figure, OPLSSA received competitive rankings on all cases, which proves that the overall performance of the OPLSSA algorithm outperforms its competitors.

#### 4.1.2 Comparison against other swarm intelligent algorithms

In this subsection, the developed OPLSSA algorithm was compared with marine predators algorithm (MPA) (Faramarzi et al., 2020a), hunger game search (HGS) algorithm (Yang et al., 2021), Improved grey wolf optimization (IGWO) algorithm (Nadimi-Shahraki et al., 2021), tunicate swarm algorithm (TSA) (Kaur et al., 2020), adaptive moth flame optimizer (WEMFO) (Shan et al., 2021), Archimedes optimization algorithm (AOA) (Hashim et al., 2021), OBL-based grey wolf optimization (OGWO) algorithm (Yu et al., 2021), diversity and mutation mechanisms enhanced moth-flame optimization (DMMFO) algorithm (Ma et al., 2021). In this comparison experiment, the 23 benchmark problems in Table 2 were employed, and the dimensions of the problems were set to 100. The parameters of the methods were set identically as in Subsection 4.1.1. Each algorithm was run 30 times independently on each function to make the obtained results more reliable. Table 5 shows the average and standard deviation of the optimal objective values obtained from the 30 executions. At the bottom of Table 5, the average ranking of the algorithms involved is presented. Table 6 reports the results gained from the Wilcoxon signed ranks test.

**TABLE 5:** Comparisons of nine algorithms on 23 test functions with 100 dimensions.

Function	Results	TSA	MPA	HGS	AOA	IGWO	WEMFO	DMMFO	OGWO	OPLSSA
f1	Mean	3.01E-10	1.80E-19	2.23E-152	9.49E-79	2.21E-12	3.59E-22	3.29E+04	4.59E-15	0
	Std	3.25E-10	1.27E-19	1.22E-151	5.09E-78	1.32E-12	1.93E-21	7.03E+03	5.59E-15	0
	f-rank	8	5	2	3	7	4	9	6	1
f2	Mean	1.41E-10	7.00E-20	8.26E-149	8.91E-78	1.09E-12	1.68E-22	1.48E+04	9.72E-16	0
	Std	1.57E-10	6.87E-20	4.52E-148	4.59E-77	1.41E-12	8.42E-22	2.65E+03	1.36E-15	0
	f-rank	8	5	2	3	7	4	9	6	1
f3	Mean	1.28E+04	9.6728	3.73E-18	1.14E-62	5.69E+03	4.63E-07	2.37E+05	8.28E+02	0
	Std	7.98E+03	1.22E+01	2.04E-17	5.34E-62	3.15E+03	2.53E-06	4.31E+04	1.01E+03	0
	f-rank	8	5	3	2	7	4	9	6	1
f4	Mean	5.59E+01	1.87E-07	4.79E-62	3.47E-38	5.0967	2.04E-10	8.89E+01	1.8357	0
	Std	1.34E+01	8.76E-08	2.62E-61	1.09E-37	3.2803	4.26E-10	2.6732	1.9532	0
	f-rank	7	5	2	3	8	4	9	6	1
f5	Mean	1.34E+01	0	0	0	0	0	3.53E+04	0	0
	Std	9.6264	0	0	0	0	0	6.17E+03	0	0
	f-rank	8	1	1	1	1	1	9	1	1
f6	Mean	4.99E-18	8.77E-41	8.25E-261	4.89E-164	1.72E-22	7.39E-46	1.02E+20	3.51E-28	0
	Std	2.62E-17	1.44E-40	0	0	2.97E-22	4.03E-45	2.86E+01	7.99E-28	0
	f-rank	8	5	2	3	7	4	9	6	1
f7	Mean	5.22E-02	2.00E-03	1.90E-03	7.68E-04	1.44E-02	1.60E-03	9.24E+01	2.10E-03	4.87E-05
	Std	1.70E-02	1.00E-03	2.50E-03	5.45E-04	5.40E-03	1.10E-03	2.96E+01	1.90E-03	5.19E-05
	f-rank	8	5	4	2	7	3	9	6	1
f8	Mean	1.83E-42	4.25E-60	1.52E-77	7.57E-187	2.57E-57	1.66E-82	1.40E-03	1.26E-61	0
	Std	9.92E-42	1.12E-59	8.29E-77	0	1.31E-56	6.92E-82	1.60E-03	6.84E-51	0
	f-rank	8	5	4	2	6	3	9	7	1
f9	Mean	7.98E-07	9.23E-16	3.30E-160	4.07E-74	3.60E-09	6.95E-19	2.07E+08	4.56E-12	0
	Std	1.30E-06	9.36E-16	1.81E-159	1.87E-73	2.68E-09	2.24E-18	9.88E+07	4.89E-12	0
	f-rank	8	5	2	3	7	4	9	6	1
f10	Mean	4.28E-02	8.66E-39	1.95E-54	5.97E-170	4.60E-10	2.88E-48	2.86E+17	5.41E-21	0
	Std	1.11E-01	1.67E-38	1.03E-53	0	1.69E-09	8.27E-48	1.19E+17	1.21E-20	0
	f-rank	8	5	3	2	7	4	9	6	1
f11	Mean	7.21E-20	3.89E-53	1.62E-43	4.26E-183	6.04E-28	8.17E-63	9.86E-01	2.85E-38	0
	Std	2.95E-19	7.53E-53	8.76E-43	0	1.43E-27	4.09E-62	3.91E-01	9.84E-38	0
	f-rank	8	4	5	2	7	3	6	9	1
f12	Mean	9.86E+02	0	0	0	1.46E+02	2.85E+02	8.14E+02	1.1484	0
	Std	1.04E+02	0	0	0	4.72E+01	3.14E+02	6.94E+01	2.7139	0
	f-rank	9	1	1	1	6	7	8	5	1
f13	Mean	1.25E-05	5.03E-11	8.88E-16	1.86E+01	1.53E-07	6.67E-01	1.97E+01	4.91E-09	8.88E-16
	Std	2.86E-05	3.14E-11	0	5.0657	6.66E-08	3.6445	3.34E-01	1.57E-09	0
	f-rank	6	3	1	8	5	7	9	4	1
f14	Mean	1.63E-02	0	0	0	4.70E-03	0	3.02E+02	1.80E-03	0
	Std	1.80E-02	0	0	0	7.10E-03	0	5.85E+01	7.00E-03	0
	f-rank	8	1	1	1	7	1	9	6	1
f15	Mean	1.51E+02	3.83E-12	9.59E-72	6.71E-42	3.70E-03	5.58E+01	5.79E+01	2.05E-04	0
	Std	2.16E+01	3.34E-12	5.25E-71	2.71E-41	1.90E-03	2.67E+01	1.01E+01	4.99E-04	0
	f-rank	9	4	2	3	6	7	8	5	1
f16	Mean	1.38E+02	1.83E-16	5.31E-123	3.44E-61	8.2595	5.44E-21	1.56E+03	2.97E-23	0
	Std	9.02E+01	4.55E-16	2.91E-122	1.88E-60	4.4488	2.95E-20	2.13E+02	1.37E-22	0
	f-rank	8	6	2	3	7	5	9	4	1
f17	Mean	3.2947	0	0	0	2.63E-14	0	1.26E+01	5.39E-15	0
	Std	2.8236	0	0	0	8.78E-15	0	1.6209	2.58E-15	0
	f-rank	8	1	1	1	7	1	9	6	1
f18	Mean	9.89E-12	2.99E-19	1.49E-109	2.08E-69	1.72E-12	7.12E-23	5.07E+02	6.62E-16	2.38E-44
	Std	1.45E-11	3.67E-19	8.17E-109	1.14E-68	1.89E-12	3.85E-22	7.89E+01	5.22E-16	4.48E-15
	f-rank	8	5	1	2	7	4	9	6	3
f19	Mean	1.09E+01	7.27E-22	4.13E-136	7.49E-81	1.61E-14	4.56E-27	1.62E+02	5.65E-27	0
	Std	5.97E+01	1.00E-21	2.26E-135	3.45E-80	1.43E-14	1.28E-26	3.67E+01	2.26E-26	0
	f-rank	8	6	2	3	7	4	9	5	1
f20	Mean	2.8523	4.58E-07	2.19E-39	8.39E-22	1.02E-02	2.04E-08	8.4517	7.17E-05	0
	Std	1.4825	4.09E-07	8.26E-39	2.07E-21	3.10E-03	4.15E-08	3.23E-01	4.19E-05	0
	f-rank	8	5	2	3	7	4	9	6	1
f21	Mean	4.3276	0	0	0	2.16E-12	0	2.19E+03	2.95E-15	0
	Std	1.21E+01	0	0	0	1.55E-12	0	4.97E+02	3.28E-15	0
	f-rank	8	1	1	1	7	1	9	6	1
f22	Mean	7.2165	6.13E-01	3.63E-38	1.71E-02	3.0308	4.60E-03	7.4324	8.31E-01	0
	Std	5.16E-01	7.47E-02	1.99E-37	1.78E-02	4.72E-01	6.80E-03	2.66E-01	2.58E-01	0
	f-rank	8	5	2	4	7	3	9	6	1
f23	Mean	8.46E-14	3.86E-55	6.3951	6.12E-235	6.93E-25	1.58E-70	6.23E-01	3.89E-64	0
	Std	2.86E-13	1.06E-54	8.6601	0	2.46E-24	8.54E-70	2.75E-01	2.12E-63	0
	f-rank	7	5	9	2	6	3	8	4	1
	Average f-rank	7.9130	4.0435	2.3913	2.5217	6.5217	3.6956	8.7391	5.5612	1.0869
	Overall f-rank	8	5	2	3	7	4	9	6	1

**TABLE 6:** Statistical conclusions based on Wilcoxon signed-rank test on 100-dimensional benchmark problems.

Function	TSA *p*-value	MPA *p*-value	HGS *p*-value	AOA *p*-value	IGWO *p*-value	WEMFO *p*-value	DMMFO *p*-value	OGWO *p*-value
f1	1.2118E-12	1.2118E-12	1.2118E-12	1.2118E-12	1.2118E-12	1.2118E-12	1.2118E-12	1.2118E-12
f2	1.2118E-12	1.2118E-12	6.6167E-04	4.5736E-12	1.2118E-12	1.2118E-12	1.2118E-12	1.9346E-10
f3	1.2118E-12	1.2118E-12	8.8658E-07	1.2118E-12	1.2118E-12	1.2118E-12	1.2118E-12	1.2118E-12
f4	1.2118E-12	1.2118E-12	3.4526E-07	1.2118E-12	1.2118E-12	1.2118E-12	1.2118E-12	1.2118E-12
f5	4.5342E-12	N/A	N/A	N/A	N/A	N/A	1.2118E-12	N/A
f6	3.0199E-11	3.0199E-11	1.0702E-09	3.0199E-11	3.0199E-11	3.0199E-11	3.0199E-11	2.3715E-10
f7	3.0199E-11	3.3384E-11	2.1947E-08	2.3715E-10	3.0199E-11	4.9752E-11	3.0199E-11	1.7769E-10
f8	1.2118E-12	1.2118E-12	8.87E-07	1.2118E-12	1.2118E-12	1.2118E-12	1.2118E-12	1.2118E-12
f9	1.2118E-12	1.2118E-12	6.6167E-04	1.2118E-12	1.2118E-12	1.2118E-12	1.2118E-12	1.2118E-12
f10	1.2118E-12	1.2118E-12	5.3750E-06	1.2118E-12	1.2118E-12	1.2118E-12	1.2118E-12	1.2118E-12
f11	1.2118E-12	1.2118E-12	5.3750E-06	1.2118E-12	1.2118E-12	1.2118E-12	1.2118E-12	1.2118E-12
f12	1.2118E-12	N/A	N/A	N/A	1.2118E-12	2.2130E-06	1.2118E-12	1.2108E-12
f13	1.2118E-12	1.2118E-12	N/A	1.6572E-11	1.2118E-12	4.5736E-12	1.2118E-12	1.2118E-12
f14	1.2118E-12	N/A	N/A	N/A	1.2118E-12	N/A	1.2118E-12	1.2118E-12
f15	1.2118E-12	1.2118E-12	3.3149E-04	1.2118E-12	1.2118E-12	1.2118E-12	1.2118E-12	1.2118E-12
f16	1.2118E-12	1.2118E-12	5.3750E-06	1.2118E-12	1.2118E-12	1.2118E-12	1.2118E-12	1.2118E-12
f17	1.2118E-12	N/A	N/A	N/A	1.1010E-12	N/A	1.2118E-12	4.4162E-11
f18	3.0199E-11	3.0199E-11	2.3982E-11	3.0199E-11	3.0199E-11	3.0199E-11	3.0199E-11	3.0199E-11
f19	1.2118E-12	1.2118E-12	1.2717E-05	1.2118E-12	1.2118E-12	1.2118E-12	1.2118E-12	1.2118E-12
f20	1.2118E-12	1.2118E-12	5.5843E-03	1.2118E-12	1.2118E-12	1.2118E-12	1.2118E-12	1.2118E-12
f21	1.2118E-12	N/A	N/A	N/A	1.2118E-12	N/A	1.2118E-12	1.2029E-12
f22	1.2118E-12	1.1037E-12	1.4552E-04	1.2118E-12	1.2118E-12	1.2000E-12	6.4999E-13	1.2118E-12
f23	1.2118E-12	1.2118E-12	1.2118E-12	4.5736E-12	1.2118E-12	1.2118E-12	1.2118E-12	1.2118E-12
+/ = /-	23/0/0	18/5/0	16/6/1	18/5/0	22/1/0	19/4/0	23/0/0	22/1/0

King, OPLSSA gets the highest rank, followed by HGS, AOA, WEMFO, MPA, OGWO, IGWO, TSA, DMMFO, which further indicates that the performance of OPLSSA is better than its competitors.

From Table 5, OPLSSA beats TSA and DMMFO on all cases. Compared to MPA, OPLSSA finds similar and better values on five and 18 test functions, respectively. HGS and OPLSSA reach the theoretical optimal solution on six benchmarks (i.e. *f*_5_, *f*_12_, *f*_13_, *f*_14_, *f*_17_, *f*_21_); OPLSSA shows better performance on 16 cases; on the remaining one function (i.e. *f*_18_), the OPLSSA algorithm is inferior to HGS. According to the pairwise comparison between OPLSSA and AOA, they are tied on five test functions; OPLSSA wins on 17 test cases; AOA gains advantage on only one function (i.e. *f*_18_). OPLSSA beats IGWO and OGWO on almost all benchmark functions; for *f*_5_, all three methods find the theoretical optimal solution. With respect to WEMFO, OPLSSA obtains similar and better results on four and 19 problems, respectively. According to the average ran-

Finally, from the results generated by the Wilcoxon signed ranks test, the *p*-values derived from all available comparisons are less than 0.05, which reveals that all differences between the performance of OPLSSA and its competitors on the utilized functions are statistically significant.

**FIGURE 6:**
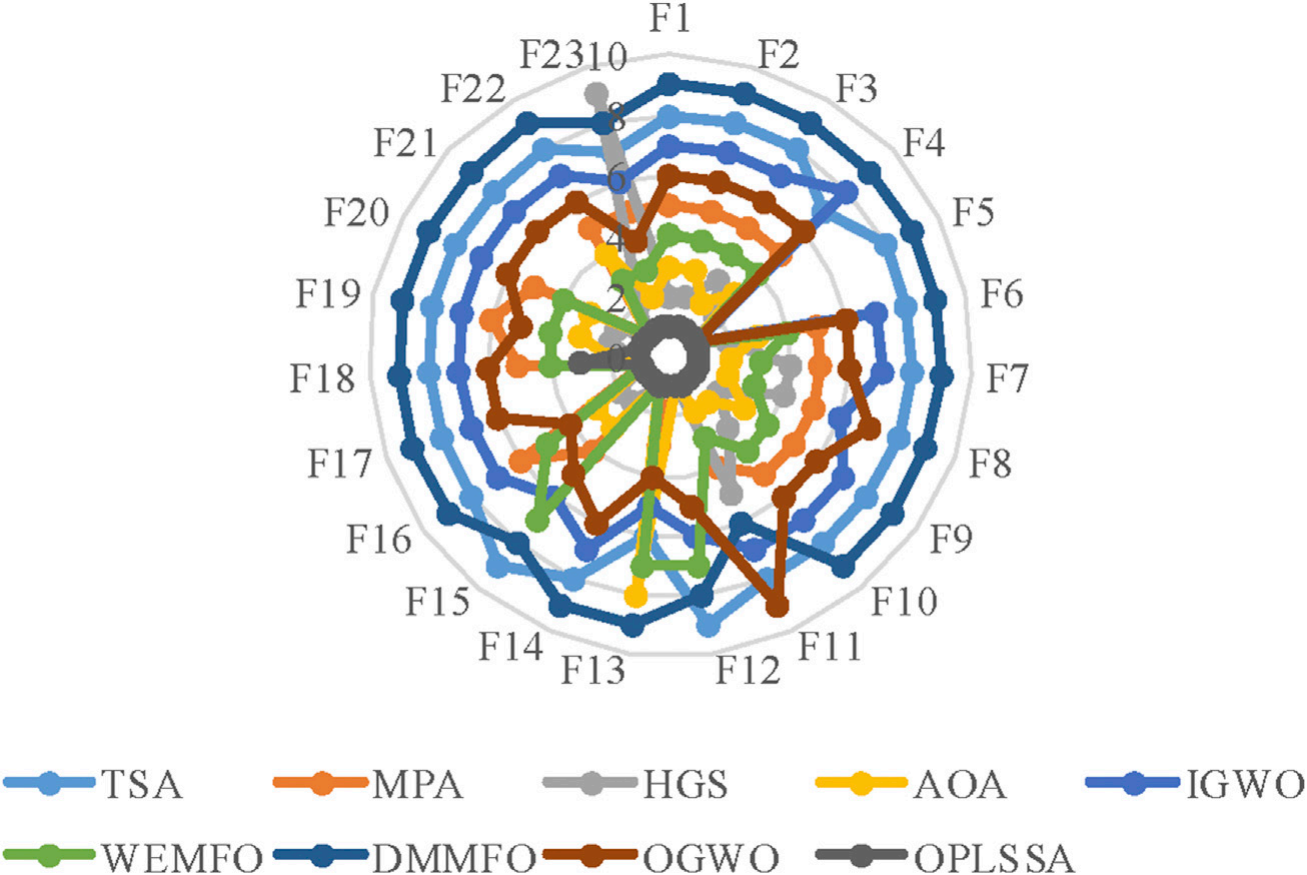
Radar plot for consolidated ranks of 23 benchmark problems with OPLSSA and the Frontier algorithms.

Figure 6 provides a graphical depiction in the form of a radar chart that emphasizes the average ranking of the OPLSSA approach and the eight involved Frontier swarm intelligent algorithms on the 23 tested functions. From the figure, OPLSSA achieved the highest ranking on almost all tested functions, which represents that this algorithm can be considered as a promising optimization tool.

#### 4.1.3 Scalability test

The performance of well-established metaheuristic algorithms will not deteriorate drastically as the dimensionality of the to-be-solved problem increases. The proposed OPLSSA algorithm aims to improve the overall performance of the basic SSA, and scalability is a key point that must be considered. In this experiment, OPLSSA was applied to address 23 benchmark functions with large scales (i.e. 10000 dimensions) in Table 2. The parameters of the algorithms were set to the same as those used in Subsection 4.1.1. To evaluate the performance of the OPLSSA method in solving challenging optimization problems with high dimensions, a novel metric called success rate (SR%) was introduced, which can be defined as

$$\left\{\begin{array}{l} {\left|f_{A}-f_{T}\right|/f_{T}\leq 10^{-5},\;f}_{T}=0\\ \left|f_{A}-f_{T}\right|\leq 10^{-5}\leq 10^{-5},f_{T}\neq 0 \end{array}\right.$$ (10)

where *f*_*A*_ is the results achieved by OPLSSA for the test function, *f*_*T*_ stand for the theoretical optimal value of the function.

If the result obtained by the algorithm on the benchmark function satisfies Eq. 10, it means that this solution is successful and *vice versa*. The OPLSSA algorithm is run 30 times independently on each case and the ratio of the number of successes to the total number of runs is the SR%. Table 7 reports the optimal value, worst value, average value, standard deviation, and SR% for the 30 independent runs.

**TABLE 7:** Results obtained by OPLSSA on 10000-dimensional functions.

Function	OPLSSA				
	Best	Worst	Mean	Std	SR%
f1	0	0	0	0	100
f2	0	0	0	0	100
f3	0	0	0	0	100
f4	0	0	0	0	100
f5	0	0	0	0	100
f6	0	0	0	0	100
f7	4.63E-07	2.95E-04	8.56E-05	8.48E-05	70
f8	0	0	0	0	100
f9	0	0	0	0	100
f10	0	0	0	0	100
f11	0	0	0	0	100
f12	0	0	0	0	100
f13	8.88E-16	8.88E-16	8.88E-16	0	100
f14	0	0	0	0	100
f15	0	0	0	0	100
f16	0	0	0	0	100
f17	0	0	0	0	100
f18	3.49E-43	6.13E-43	4.71E-43	7.40E-44	100
f19	0	0	0	0	100
f20	0	0	0	0	100
f21	0	0	0	0	100
f22	0	0	0	0	100
f23	0	0	0	0	100

Me representative test functions were plotted. In experiment 2, the convergence graphs of the OPLSSA algorithm and eight cutting-edge approaches on some representative benchmark functions were illustrated.

From Table 7, OPLSSA shows competitive performance in solving high-dimensional optimization problems. In terms of solution accuracy, OPLSSA is able to find the theoretical optimal solution on 20 test functions; for *f*_7_ and *f*_13_, the solutions obtained are similar to those achieved on 100-dimensioanl problems; for *f*_18_, the value derived is inferior to that found on 100-dimensional benchmark. This proves that the dramatic increase in the problem’s dimensionality does not deteriorate the performance of the OPLSSA algorithm, i.e., OPLSSA has superior stability. On the other hand, in terms of SR%, OPLSSA attains a 100% success rate on 22 high-dimensional optimization problems, representing that the developed approach has been successful in all 30 independent tests. The SR% on another function (i.e. *f*_7_) is 70%, meaning that 21 out of 30 runs achieved success. Based on the above discussion, the developed OPLSSA algorithm gets remarkable performance on large scale optimization problems.

#### 4.1.4 Convergence analysis

A well-established swarm intelligent algorithm moves in large steps in the search space in the early iterations to locate the rough position of the global optimal solution. After the lapse of few iterations, the step size is shortened to precisely search the already explored region, thus improving the convergence accuracy. Rapid convergence rate often leads to premature convergence of the algorithm, making the solution accuracy insufficient. Improving the solution precision of the method requires performing more iterations, which will degrade the convergence speed of the algorithm. The unbalanced convergence speed and convergence precision is a weak open point that destroys the performance of the algorithm. One of the main goals of the improvements to SSA in this work is to enhance the above-mentioned balance of the basic algorithm. To investigate the performance of the OPLSSA algorithm in this regard, two additional sets of experiments were performed. For experimental purpose, some of the representative benchmarks with 100-dimensional in Table 2 were applied. The parameter settings of the employed algorithms were the same as those used in Subsection 4.1.1. In experiment 1, the convergence curves of the standard SSA algorithm and eight SSA variants on so-

**FIGURE 7:**
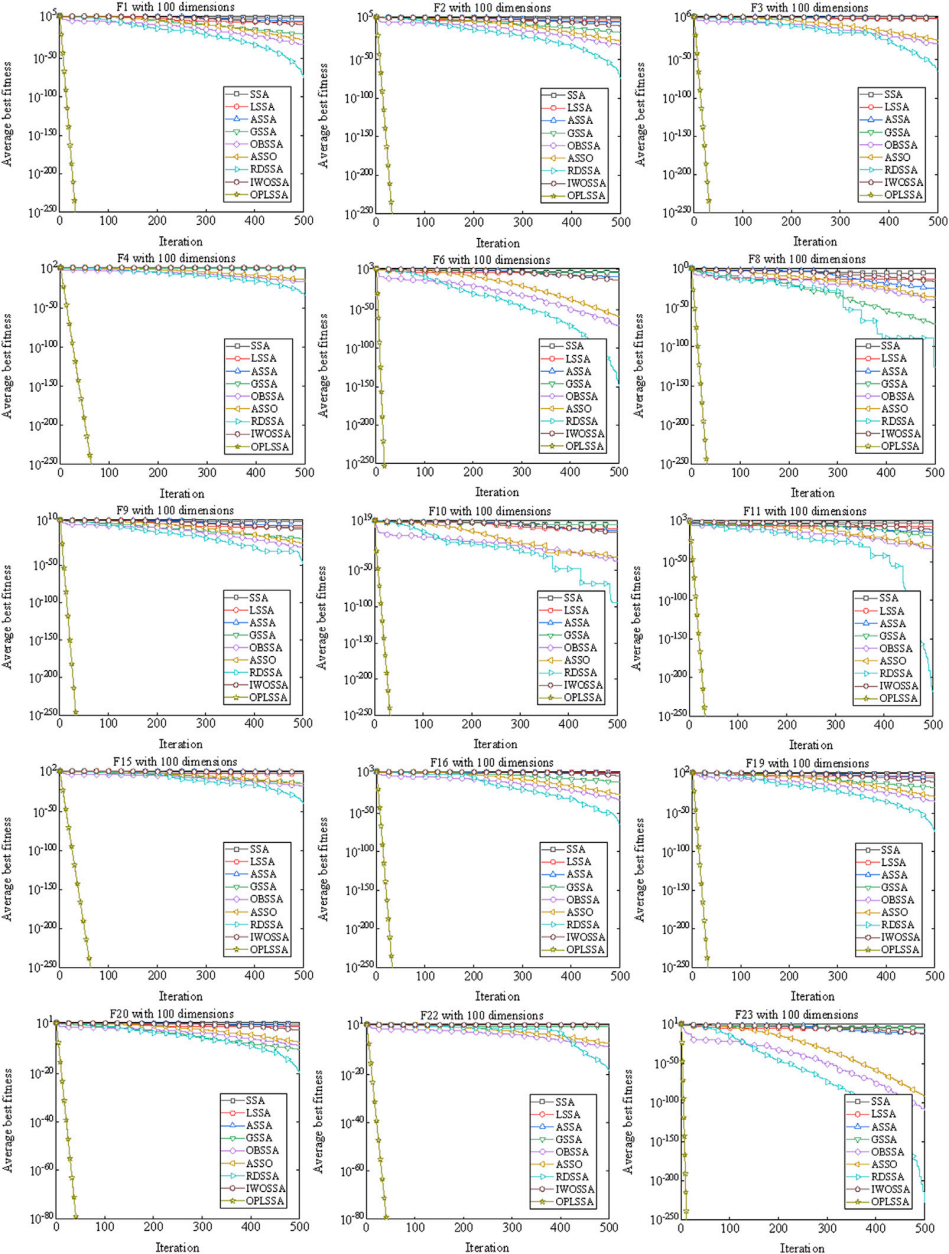
Convergence curves of OPLSSA and other SSA-based algorithms on 15 representative benchmarks.

**FIGURE 8:**
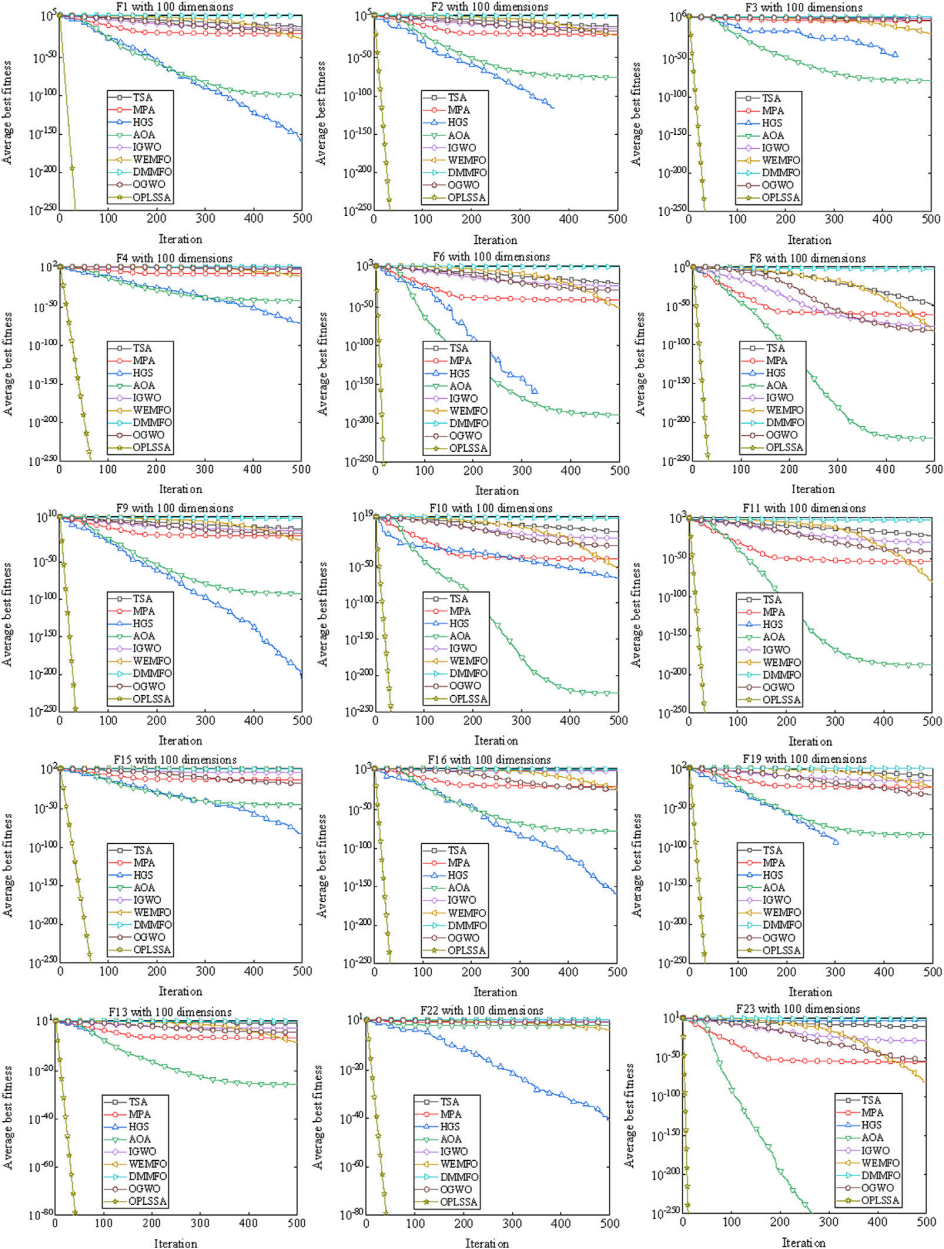
Convergence curves of OPLSSA and other Frontier algorithms on 15 representative benchmarks.

By observing Figure 7, the OPLSSA algorithm is able to find more accurate solutions quickly for all functions, while the comparison algorithms are inferior to OPLSSA in terms of convergence rate and convergence accuracy. Similar phenomena can be observed on Figure 8. Overall, the OPLSSA algorithm outperforms the popular SSA variants and the cutting-edge algorithms in terms of convergence rate and convergence accuracy.

### 4.2 Experiments on CEC 2017 benchmark functions

In this subsection, the effectiveness of OPLSSA algorithm is tested on the IEEE CEC 2017 benchmark functions. This benchmark suite contains 30 test problems. For each function, the search region in each dimension is defined as [-100, 100]. All selected functions are initiated in [-100, 100]^*D*^, where *D* is the problem’s dimension which is taken as 30 for all functions. All the 30 benchmarks can be classified into four species according to the function character, in which unimodal functions contain F1, F2, and F3, from F4 to F10 belongs to multimodal functions, hybrid functions include from F11 to F20, from F21 to F30 belongs to composition functions. The details of all the 30 benchmark functions are shown in Table 8. Since the mentioned functions are from distinct classes and each has its own characteristic properties, the utilized OPLSSA is challenged from different aspects. The obtained results are compared with other well-established swarm intelligent approaches, including DA (Mirjalili, 2016), TSA (Kaur et al., 2020), EGWO (Long et al., 2018), GOA (Abualigah and Diabat, 2020), SOA (Wang et al., 2022b), PBO (Polap and Woźniak, 2017), and the standard SSA (Mirjalili et al., 2017). The specific parameters of the comparison approaches are set the same as recommended in the respective original literature, and the number of function evaluations is set to 10^4^×*D*, where *D* is the dimension of the tested problem. The results obtained at CEC 2017 test functions for the involved approaches are reported in Table 9. Note that F2 has been removed due to the erratic behavior it exhibits.

**TABLE 8:** Summary of the 30 CEC 2017 benchmark problems.

Class	No.	Description	Search Range	Optimal
Unimodal	1	Shifted and Rotated Bent Cigar Function	[−100, 100]	100
	2	Shifted and Rotated Sum of Different Power Function	[−100, 100]	200
	3	Shifted and Rotated Zakharov Function	[−100, 100]	300
Multimodal	4	Shifted and Rotated Rosenbrock’s Function	[−100, 100]	400
	5	Shifted and Rotated Rastrigin’s Function	[−100, 100]	500
	6	Shifted and Rotated Expanded Scaffer’s Function	[−100, 100]	600
	7	Shifted and Rotated Lunacek Bi-Rastrigin Function	[−100, 100]	700
	8	Shifted and Rotated Non-Continuous Rastrigin’s Function	[−100, 100]	800
	9	Shifted and Rotated Levy Function	[−100, 100]	900
	10	Shifted and Rotated Schwefel’s Function	[−100, 100]	1000
Hybrid	11	Hybrid Function 1 (N = 3)	[−100, 100]	1100
	12	Hybrid Function 2 (N = 3)	[−100, 100]	1200
	13	Hybrid Function 3 (N = 3)	[−100, 100]	1300
	14	Hybrid Function 4 (N = 4)	[−100, 100]	1400
	15	Hybrid Function 5 (N = 4)	[−100, 100]	1500
	16	Hybrid Function 6 (N = 4)	[−100, 100]	1600
	17	Hybrid Function 6 (N = 5)	[−100, 100]	1700
	18	Hybrid Function 6 (N = 5)	[−100, 100]	1800
	19	Hybrid Function 6 (N = 5)	[−100, 100]	1900
	20	Hybrid Function 6 (N = 6)	[−100, 100]	2000
Composition	21	Composition Function 1 (N = 3)	[−100, 100]	2100
	22	Composition Function 2 (N = 3)	[−100, 100]	2200
	23	Composition Function 3 (N = 4)	[−100, 100]	2300
	24	Composition Function 4 (N = 4)	[−100, 100]	2400
	25	Composition Function 5 (N = 5)	[−100, 100]	2500
	26	Composition Function 6 (N = 5)	[−100, 100]	2600
	27	Composition Function 7 (N = 6)	[−100, 100]	2700
	28	Composition Function 8 (N = 6)	[−100, 100]	2800
	29	Composition Function 9 (N = 3)	[−100, 100]	2900
	30	Composition Function 10 (N = 3)	[−100, 100]	3000

**TABLE 9:** Results of CEC 2017 at 30-dimensional achieved by the developed algorithm and its competitors.

Function	DA	GOA	TSA	SOA	EGWO	PBO	SSA	OPLSSA
F1	2.04E+08	1.03E+09	1.60E+10	7.37E+09	5.85E+09	1.36E+04	6.34E+02	1.06E+02
F2	NA	NA	NA	NA	NA	NA	NA	NA
F3	5.43E+04	3.09E+04	4.92E+04	2.98E+04	5.28E+04	1.08E+04	1.55E+04	3.00E+02
F4	7.76E+02	4.89E+02	3.36E+03	7.15E+02	1.33E+04	5.46E+02	5.15E+02	4.69E+02
F5	7.67E+02	7.17E+02	8.14E+02	6.90E+02	9.43E+02	6.80E+02	6.15E+02	5.66E+02
F6	6.65E+02	6.32E+02	6.63E+02	6.35E+02	7.04E+02	6.63E+02	6.24E+02	6.03E+02
F7	1.01E+03	8.20E+02	1.23E+03	1.08E+03	1.43E+03	1.79E+03	8.37E+02	8.05E+02
F8	1.04E+03	1.04E+03	1.05E+03	9.48E+02	1.20E+03	9.48E+02	9.14E+02	8.47E+02
F9	9.31E+03	4.44E+03	8.69E+03	3.63E+03	1.58E+04	3.92E+03	3.00E+03	9.19E+02
F10	6.54E+03	5.07E+03	6.64E+03	6.44E+03	9.83E+03	4.21E+03	3.83E+03	2.98E+03
F11	4.34E+03	9.21E+03	4.35E+03	1.88E+03	9.16E+03	1.26E+03	1.21E+03	1.14E+03
F12	8.31E+07	4.87E+06	2.10E+09	2.20E+08	1.67E+09	6.52E+06	1.44E+06	3.85E+05
F13	3.73E+05	2.82E+04	1.13E+09	1.48E+08	3.65E+09	1.80E+05	5.05E+04	1.30E+04
F14	1.46E+05	8.32E+03	1.45E+06	1.17E+05	4.16E+05	1.91E+04	3.92E+03	1.92E+03
F15	4.98E+04	1.86E+05	2.52E+07	1.51E+05	8.07E+07	3.45E+04	1.01E+04	8.98E+03
F16	3.39E+03	3.42E+03	2.94E+03	2.65E+03	7.26E+03	2.72E+03	2.46E+03	1.96E+03
F17	2.80E+03	2.13E+03	2.42E+03	2.06E+03	5.42E+03	2.26E+03	1.97E+03	1.80E+03
F18	2.81E+06	8.82E+04	1.34E+06	4.42E+05	1.19E+07	1.52E+05	1.91E+05	2.95E+04
F19	8.46E+06	4.35E+06	7.80E+06	5.04E+06	3.09E+08	4.82E+05	1.27E+05	9.51E+03
F20	3.02E+03	2.91E+03	2.61E+03	2.63E+03	3.55E+03	2.32E+03	2.32E+03	2.11E+03
F21	2.60E+03	2.51E+03	2.59E+03	2.45E+03	2.77E+03	2.59E+03	2.39E+03	2.36E+03
F22	3.13E+03	2.49E+03	7.68E+03	2.66E+03	7.66E+03	6.24E+03	2.30E+03	2.30E+03
F23	3.37E+03	2.90E+03	3.16E+03	2.82E+03	3.82E+03	3.31E+03	2.74E+03	2.69E+03
F24	3.56E+03	3.01E+03	3.31E+03	2.93E+03	4.09E+03	3.42E+03	2.89E+03	2.90E+03
F25	2.98E+03	2.89E+03	3.42E+03	3.23E+03	5.56E+03	2.88E+03	2.89E+03	2.88E+03
F26	8.75E+03	4.21E+03	7.70E+03	5.09E+03	1.22E+04	7.88E+03	5.42E+03	4.11E+03
F27	3.32E+03	3.29E+02	3.43E+03	3.25E+03	6.03E+03	3.20E+03	3.24E+03	3.19E+03
F28	3.53E+03	4.43E+03	4.49E+03	5.29E+03	7.35E+03	3.30E+03	3.22E+03	3.10E+03
F29	4.85E+03	4.22E+03	4.73E+03	4.01E+03	7.75E+03	4.81E+03	3.83E+03	3.48E+03
F30	1.63E+07	1.93E+06	1.89E+07	1.09E+07	2.48E+09	1.28E+06	3.24E+06	3.02E+05

By observing Table 9, OPLSSA outperformed DA, GOA, TSA, SOA, EGWO and PBO on all test cases. OPLSSA can beat PBO on almost all test functions, while on F22 and F24, the two algorithms obtain similar performance. With respect to SSA, OPLSSA provides better results on 27 test functions and similar values on one case. However, for F24, marginally better results are achieved by SSA. Overall, OPLSSA shows better or at least competitive performance than its peers on all CEC 2017 test functions, which proves that OPLSSA has superior performance. Moreover, according to the pairwise comparisons between SSA and DA, GOA, TSA, SOA, EGWO and PBO, SSA beats all competitors on 20 test functions (i.e. F1, F5, F6, F8, F9, F10, F11, F12, F14, F15, F16, F17, F19, F20, F21, F22, F23, F24, F28, F29), which proves that the SSA algorithms is a competitive swarm intelligence-based approach.

## 5 Conclusion

This paper proposes an extended version of salp swarm algorithm termed as OPLSSA. Two modifications to SSA have been introduced which make it competitive with other well-established swarm intelligent algorithms: First, the algorithm applied the PIBL mechanism to help the leading salp to jump out of the local optimal. Second, the algorithm uses the concept of adaptive-based mechanism to generate diversity among the followers. Both these modifications helping in boosting the balance between exploration and exploitation. The performance of the proposed algorithm has been tested on 23 classical benchmark functions and 30 IEEE CEC 2017 benchmark suite and compared with several metaheuristic techniques, including SSA-based algorithms and state-of-the-art swarm intelligent algorithms. The experimental results show that OPLSSA performs better or at least comparable to the competitor methods. Therefore, the developed OPLSSA algorithm can be regarded as a promising method for global optimization problems.

In the future works, we have planned to further extend the research on this paper on the following points: for one direction, the two proposed mechanisms will be combined with other swarm intelligence based algorithms with the hope of improving their performance; for another, the proposed OPLSSA algorithm will be employed to resolve real-world problems such as feature selection, PV parameter extraction, mobile robot path planning, multi-threshold image segmentation, and video coding optimization.

## Data Availability

The original contributions presented in the study are included in the article/supplementary material, further inquiries can be directed to the corresponding author.
